# Mindfulness, Trust, and Leader Effectiveness: A Conceptual Framework

**DOI:** 10.3389/fpsyg.2019.01588

**Published:** 2019-07-10

**Authors:** Yvonne Stedham, Theresa B. Skaar

**Affiliations:** ^1^Department of Managerial Sciences, University of Nevada, Reno, Reno, NV, United States; ^2^Interdisciplinary Social Psychology Ph.D. Program, University of Nevada, Reno, Reno, NV, United States

**Keywords:** mindfulness, mindfulness mechanisms, influence, trust, trustworthiness leader effectiveness, leadership

## Abstract

In this conceptual research, the authors develop an integrative framework for the relationship between mindfulness and leader effectiveness. Mindfulness is suggested to affect leader effectiveness via its impact on leader characteristics and behaviors that enable trust based relationships between the leader and followers. The paper provides detailed overviews of trust and mindfulness and their relationship to leadership. In-depth theoretical reflections on the linkages among the relevant concepts are offered. Of particular interest are the relationships between mindfulness mechanisms, leader characteristics and behaviors, and trustworthiness and trust.

## Introduction

Leadership is about influencing others’ behaviors (Bass, 1960; Avolio et al., 2004; Brown et al., 2005; Goleman, 2013). In fact, a leader’s ideas and vision become reality only through the actions of others. Leadership has been studied by academics for more than a century. Early models of leadership have been characterized as transactional and focus on leader characteristics and behaviors and the associated transactions with followers (Bass and Bass, 2008). More recent research suggests that leadership is about understanding and relating to the followers (George, 2003; Avolio et al., 2004; Northouse, 2016) and investigates the relationship between the person who leads and the individuals who choose to follow that person. The resulting models imply that individuals only choose to follow someone and allow that person to influence them if they trust that person (e.g., Bass and Riggio, 2006; Bass and Bass, 2008; Wang and Hsieh, 2013). In this paper, we propose that mindfulness facilitates a person’s ability to engage in behaviors that create trusting relationships and by that enhance leader effectiveness.

Mindfulness is a state of being openly attentive to and aware of what is taking place in the present moment, internally and externally (Kabat-Zinn, 1982, 1990; Brown and Ryan, 2003). The impact of practicing mindfulness on self- and social awareness and on self-regulation of emotions and behaviors has been well documented (e.g., Goleman and Davidson, 2017). Mindfulness has been found to have beneficial effects on physical and psychological well-being. Empirical results demonstrate, for example, the positive impact of mindfulness on stress management (e.g., Shapiro et al., 2005; Chiesa and Serretti, 2009), dealing with addiction (Brewer, 2017), anxiety (e.g., Tacón et al., 2003), and Post Traumatic Stress Disorder (PTSD) (e.g., Boden et al., 2012; Owens et al., 2012). Within the workplace,mindfulness has been shown to relate positively to a range of performance categories (Shao and Skarlicki, 2009; Dane and Brummel, 2014; Reb et al., 2014), interpersonal relationships (Beckman et al., 2012; Beach et al., 2013; Reb et al., 2014; Verdorfer, 2016), and employee well-being (Danna and Griffin, 1999; Eberth and Sedlmeier, 2012).

Surprisingly, few studies have focused on the relationship between mindfulness and leadership or leader effectiveness. Good et al. (2016, p. 127) comment that “despite its importance to management, leadership has not been extensively studied by mindfulness researchers” and “that the focus has not been directly on the relationship between leaders and followers but rather on the beneficial role of individual mindfulness for leaders and followers.” Similarly, Pinck and Sonnentag (2018) point out that very little research has addressed how leader mindfulness impacts employee well-being and Reb et al. (2014) explicitly call for research that sheds light on the relationship between leader mindfulness and existing leadership constructs.

A number of leadership researchers have identified trust as an essential factor in leadership and leader effectiveness (Atwater, 1988; Posner and Kouzes, 1993; Brower et al., 2000; Jung and Avolio, 2000; Bass and Bass, 2008). However, very little is known about the relationship between specific leader behaviors and trust and leader effectiveness. In this paper, we integrate prior research on mindfulness in the workplace and on trust and leadership and offer a conceptual framework proposing that mindfulness impacts leader effectiveness via its impact on leader characteristics and behaviors that enable trust-based relationships between the leader and followers.

We organize this article as follows: We begin with an overview of what mindfulness is, explain how it works, and provide a brief review of the literature on the relationship between mindfulness and leadership. Next, we discuss trust and its relationship to leadership and leader effectiveness. We then explore the potential relationships between mindfulness, trust, and leadership and present a conceptual framework of such relationships.

## Mindfulness: What Is It and How Does It Work?

Mindfulness techniques are now recognized as having the potential to transform workplaces (Reb and Atkins, 2015). The impact of mindfulness on performance, relationships, and well-being in organizations has been documented (Good et al., 2016). Some limited attention has recently been given to the role of mindfulness in leadership (e.g., Brown et al., 2007; Verdorfer, 2016). We suggest that mindfulness affects leadership because mindful leaders engage in behaviors that support the creation of trusting relationships.

### What Is Mindfulness: An Overview

A commonly accepted definition of mindfulness is: a mental state characterized by nonjudgmental awareness of present moment experience, including one’s sensations, thoughts, bodily states, consciousness, and the environment, while encouraging openness, curiosity, and acceptance (Kabat-Zinn, 2003; Bishop et al., 2004). Brown et al. (2007, p. 212) explain that “awareness is the conscious registration of stimuli, including the five physical senses, the kinesthetic senses, and the activities of the mind” and that awareness is our direct, most immediate contact with reality. When a stimulus is sufficiently strong, attention is engaged, which manifests as an initial taking notice of or turning toward the object (Nyanaponika, 1973). Sensory objects are held in focal attention only briefly until cognitive and emotional reactions to them ensue. Such reactions often include a primary appraisal of the object basically as “good,” “bad,” or “neutral,” usually in reference to the self.

Bishop et al. (2004) suggest that mindfulness can be conceptualized as consisting of two components. The first component is regulation of attention to maintain it on the immediate experience. The second component represents the attitude with which the experience is approached – specifically an attitude of neutrality, openness, acceptance, and curiosity. Mindfulness includes meta-attention, i.e., the awareness of having thoughts and of the origins of those thoughts (Carmody et al., 2009). More specifically, Hayes et al. (2011) suggest that mindfulness involves non-identification with the present moment experience, the ability to simply observe or notice one’s own emotions and thoughts without getting absorbed by them (meta-cognition), flexibly attending to the present moment, an open acceptance of the experience as is without wishing it to be different and wanting to change it, and allows for “defusion from the literality of verbal cognitions.”

Mindfulness can be conceptualized as a trait (e.g., Brown and Ryan, 2003; Baer et al., 2006) or a state (e.g., Lau et al., 2006). Much evidence exists for the effectiveness of a simple, repeatable *method*, meditation practice, for achieving mindfulness and its associated behaviors and attributes (Davidson et al., 2003; Siegel, 2010; Gunaratana, 2011). A common meditation exercise (mindfulness practice) is simple breath-focused attention meditation. Other practices include Hatha yoga, body scan, and walking meditation. Goleman and Davidson (2017) summarize the scientific evidence supporting the lasting traits that can result from a mindfulness meditation practice.

### How Does Mindfulness Work?

In the following sections, we will explain the processes underlying the impact of mindfulness on cognition, emotion, and behavior.

#### Re-perceiving

Shapiro et al. (2006) base their conceptualization of mechanisms underlying mindfulness interventions on Kabat-Zinn’s (1994, p. 4), definition of mindfulness “paying attention in a particular way: on purpose, in the present moment, and non-judgmentally” simply observing or “witnessing” objectively the contents of consciousness without getting caught in the narrative of our thoughts. They propose a model that suggests that intentionally attending with openness and non-judgmentalness leads to dis-identification or decentering and opens the possibility for a significant shift in perspective which they refer to as “re-perceiving.” They consider re-perceiving to be a meta-mechanism of action that is associated with additional direct mechanisms that lead to change in attitudes and behaviors. Specifically, re-perceiving enhances one’s capacity to take the perspective of another person and facilitates empathy. Hayes et al. (1999) describe the shift in perspective as a shift from “self as content” to “self as context.” This shift results in non-attachment, clarity, and more accurate perceptions and facilitates self-regulation, self-management, values clarification, cognitive, emotional, and behavioral flexibility, and exposure.

#### Cognitive Capacity and Flexibility

Mindfulness has been shown to increase cognitive capacity, specifically working memory capacity (e.g., Roeser et al., 2013). By being able to focus attention, cognitive capacity is not wasted on mind wandering and irrelevant narratives. The primary impact of mindfulness on cognition is through its impact on cognitive flexibility which is facilitated through re-perceiving. Rather than being constrained by automaticity or reactivity, re-perceiving allows for a “beginner’s mind.” This allows for more accurate and complete processing of information. Glomb et al. (2011) differentiate between core (e.g., re-perceiving/decentering) and secondary processes (e.g., response flexibility) of mindfulness. They propose that the resulting self-regulation of thoughts, emotions, and behaviors is the central benefit of a mindfulness practice.

#### Emotion Regulation

Emotions are the result of the evaluative assessment of observed stimuli. Emotion regulation refers to how individuals influence which emotions they have, when they have them, and how they experience and express them (Gross, 1998). Among others, Hölzel et al. (2011) found that mindfulness practice improves emotion regulation. Mindfulness allows for objectively, without judgment, observing an event and one’s emotional reaction to the event. This mindful experiential processing of the stimuli promotes more neutral evaluations and results in possible reconstruction of a negative or stressful event as beneficial, meaningful, or benign. Such reappraisal is one way in which emotion is regulated due to mindfulness.

Exposure, extinction, and reconsolidation are additional influences on emotion regulation (Hölzel et al., 2011). During mindfulness practice, one learns to turn toward rather than avoid unpleasant stimuli, including unpleasant emotions (exposure). A result of that exposure is the discovery that the unpleasant emotions are transient and pass away (extinction) and a “sense of safety or well-being” can be experienced in their place (reconsolidation). Such non-reactivity leads to unlearning of previous connections and thereby providing freedom from habitual emotional reactions.

Mindfulness practice effects extend beyond improving emotional self-regulation (Atkins and Styles, 2015). It has been suggested that mindfulness facilitates a shift from treating self-referential statements as literal truths to flexibly engaging with them and allowing for a self-as-process and self-as-perspective view of individual identity (Törneke, 2010). This type of shift is desirable in the work context as it results in more behavioral predictability and consistency and reduces uncertainty. Hölzel et al. (2011) provide a detailed summary of the neuroscientific findings related to changes in self-referential processing due to mindfulness. They show changes occur from a view of an unchanging self to one where the self becomes observable to the meditator through development of meta-awareness.

The efficacy of emotion regulation has been shown by the results of a meta-analysis on the effects of mindfulness programs. They showed that mindfulness is associated with less negative and more positive emotional tone (Eberth and Sedlmeier, 2012) and that mindfulness speeds up recovery from negative emotions (Keng et al., 2013). Both, more positive emotional tone and faster release of negativity are important to social interactions, relationships, and workplace climate.

#### Behavior Regulation

Mindfulness promotes regulation of behavior that improves well-being (Deci and Ryan, 1980; Brown and Ryan, 2003, 2004; Ryan, 2005). The observant processing of internal and external stimuli facilitates the regulation of action through “the provision of choice that is informed by abiding needs, values, and feelings and their fit with situational options and demands” (Brown et al., 2007, p. 223). Mindfulness-based awareness facilitates more flexible, adaptive responses, and contributes to the reduction of automatic, habitual, or impulsive reactions (Bishop et al., 2004; Ryan and Deci, 2004). When one acts mindfully, one’s action is based on a chosen response made possible by the creation of a mental gap between the stimulus–response connection that shapes automatic behavior. Such chosen behavior is disengaged from its usual causes (Baumeister and Sommer, 1997).

#### Self- and Social Awareness

Mindfulness is paying attention, in the moment, to internal and external stimuli, in a non-judgmental way. It is simply noticing, without attachment or aversion, what is happening in any given moment within a person – body sensations, emotions, and thoughts – and in the person’s environment. The positive impact of mindfulness on self-regulatory and self-referential processes discussed above allows such moment-to-moment open, accepting awareness, which results in increased self- and social awareness.

The relationship between mindfulness practice and self- and social awareness and focus have been well-established (e.g., Bishop et al., 2004; Walach et al., 2006; Coholic, 2011; Vago and Silbersweig, 2012). In fact, the measures for mindfulness include components that capture such awareness (e.g., Baer et al., 2004; Walach et al., 2006; Kohls et al., 2009).

## Leadership: the Role of Mindfulness

Leaders are individuals who see a need for action and change and are able to make change happen by inspiring and influencing others to engage in actions and behaviors that create a “new reality.” Effective leaders “see clearly” without distortions, are aware of their own emotions and filters, have empathy, and are able to create and manage relationships that result in community and synergy. Based on the research presented above, it is to be expected that mindfulness facilitates effective leadership.

Research on the relationship between mindfulness and leadership, although relatively scarce and eclectic, provides empirical support for this expectation. The ability to focus attention on the present moment, acting with intentionality, self-compassion and resilience, meta-cognition, and seeing clearly are the aspects of mindfulness that are found to facilitate leader effectiveness (Sauer and Kohls, 2011; Reb et al., 2015). Dunoon and Langer (2012) in their reflections on the relationship between mindfulness and leadership discuss the importance of alertness to multiple perspectives, active self-appraisal, and attentiveness to our use of language. These three factors are developed and maintained through mindfulness practice. Boyatzis (2015) presents a comprehensive summary of the relevant research. His review includes work on the role of mindfulness in building relationships (Boyatzis and McKee, 2005; Boyatzis et al., 2013), especially the importance of empathy (Decety and Michalska, 2010; Goleman, 2013). The findings by Reb et al. (2014) support the positive effect of leader mindfulness on employee job performance, job satisfaction, and need satisfaction, and reduction in emotional exhaustion.

Verdorfer (2016) investigates the relationship between mindfulness and leadership motivation and specific leader behaviors. Specifically, he examined mindfulness and its relation to servant leadership and found a positive relationship between mindfulness and the servant leadership dimensions humility, standing back, and authenticity and that mindfulness had a positive impact on humility and non-self-centered motivation to lead. Building on prior research that supported a positive relationship between leader mindfulness and employee well-being, Pinck and Sonnentag (2018) investigated the mediating role of transformational leadership (TFL) in that relationship. Their findings supported such a mediating role of TFL and showed a positive relationship between mindfulness and all facets (idealized influence/vision, inspirational communication/motivation, intellectual stimulation, individualized consideration/personal recognition) of TFL.

## Trust and Trustworthiness

### Trust Defined

Trust can be seen as the bridge between “the known” and “the unknown” – also referred to as the “trust leap” (Botsman and Rogers, 2011; Hawley, 2012; Botsman, 2017; Boser, 2018). Trust is to believe despite uncertainty (Misztal, 1998). The literature differentiates two types of trust: generalized (social/moralistic) trust and particularized (interpersonal) trust. Generalized trust is the belief that most people can be trusted (Rosenberg, 1956; Soroka et al., 2007; Uslaner, 2018) whereas particularized trust is the perception that another person is trustworthy relative to a specific task (Berg et al., 1995; Glaeser et al., 2000; Bauer and Freitag, 2018). In this research, the focus is on particularized/interpersonal trust. Note that for the purpose of simplicity, we will refer to interpersonal trust as “trust” for the remainder of the paper.

Trust represents a person’s willingness to be vulnerable, giving someone else the opportunity to inflict harm on oneself, a willingness to take risk (Mayer et al., 1995, 2007). Rousseau et al. (1998, p. 395) define trust as “a psychological state comprising the intention to accept vulnerability based upon the positive expectations of the intentions or behavior of another.” The literature on interpersonal trust differentiates between cognitive and affective trust (McAllister, 2005; Dirks and Ferrin, 2002; Yang et al., 2009; Wang et al., 2010; Yang and Mossholder, 2010). Cognitive trust represents a rational approach to reducing uncertainty and building trust. In contrast, affective trust represents trust based on the emotional bonds that might exist among people (Lewis and Weigert, 1985). This trust is rooted in beliefs that others’ behaviors are motivated by communal interests as well as self-interest.

### Components of Trustworthiness

Adopting Rousseau et al.’s (1998) definition, Mayer et al. (1995) developed an integrative, cognitive model of trust and suggested that trust between two people depends on the trustor’s perception of the trustee as trustworthy. They further propose that the trustor’s perception of the trustee’s trustworthiness depends on three factors: the trustor’s perception of the trustee as being competent (ability), acting with integrity, and being benevolent.

#### Ability

Ability includes skills, competencies, and characteristics that enable a person to have influence within a specific domain (Mayer et al., 1995). Ability might refer to technical or non-technical competencies. The perception of “ability” results in the expectation that the person can manage the task at hand. The point is that when a person is seen as capable, trust increases because perceived uncertainty and vulnerability decrease.

#### Integrity

Integrity involves the trustor’s perception that the trustee adheres to a set of principles that the trustor finds acceptable (Mayer et al., 1995). This is exhibited in consistency of the trustee’s behavior across situations and consistency between the trustee’s words and action, “walking the talk.” However, it is important to emphasize that for trust to increase, the set of principles that the trustee follows aligns well with the trustor’s values. As in the case of ability, the impact of perceived integrity operates through decreasing the trustor’s perception of uncertainty and vulnerability as the trustee’s behavior is predictable if she/he has been shown to consistently follow a set of principles.

#### Benevolence

Benevolence refers to the trustor’s belief that the trustee has the trustor’s best interests in mind. It implies that the trustee cares about the trustor and has some specific attachment to the trustor (Mayer et al., 1995). Benevolence is associated with trustors’ perceptions that the trustee is a caring, warm person who is aware of and concerned with the needs and well-being of others.

The three components of trust are independent from each other, each component contributing separately to perceptions of trust. Hence, trustworthiness is to be seen as a continuum rather than a dichotomy. Further, Schoorman et al. (2007) acknowledge the cognitive nature of their trustworthiness model and that emotions and affect might play an additional role. However, they suggest that “while emotions may create a temporary *irrationality* about the data on ability, benevolence, and integrity, after a period of time the perception would return to a rational perspective” (Schoorman et al., 2007, p. 349).

## Trust and Leadership

Although definitions of leadership are numerous and vary greatly, all, explicitly or implicitly, integrate the notion of influence. Leading is about influencing others’ behaviors. Leadership has been described as an interaction process and a relationship between individuals where one exerts more influence (the leader) than he/she is influenced (Haiman, 1951; Gerth and Mills, 1953). The leader is the group member whose influence on the group’s attitudes, performance, or decision-making greatly exceeds that of average members (Simonton, 1994). Successful leadership affects the behaviors of group members and the activities of a group (Bass and Bass, 2008).

Studies on leadership investigate “how does influence happen?” and “what attitudes and behaviors allow for differing levels of influence?” Overall, leadership research has produced a long list of factors that contribute to leader effectiveness and facilitate the necessary influence, including emotional intelligence, communication, empathy, creativity, and vision [see Bass and Bass (2008) for an extensive summary]. For example, Brown et al.’s (2005) study employed learning theory to explore how a leader’s ethics may be transferred to followers, Robertson and Barling (2013) looked at the leader’s influence on employees’ attitude toward climate change and protecting the environment, and Avolio et al. (2004) researched the link between a leader’s authenticity and follower attitudes, behaviors, and performance outcomes. Although approaches to leadership research differ greatly as to the specific factors studied, all acknowledge that leading involves interaction between individuals. In fact, it has been emphasized by many leadership scholars that leading is about relationship (Kouzes and Posner, 1987, 2002, 2012, 2013; Bass and Riggio, 2006).

Individuals follow leaders who they can trust and make them feel safe (Sinek, 2009; Kouzes and Posner, 2012). Trust in the leader involves positive expectations by the follower of the leader’s motives regarding the follower (Boon and Holmes, 1991).Trust in a leader is a follower’s belief that a leader can and will act on the basis of the leader’s words, actions, and decisions (McAllister, 2005). Deutsch (1992) points out that when trusting a leader, the follower becomes more vulnerable to the actions of the leader.

Given the importance of trust to understanding leadership, it is surprising that the literature on this topic is relatively scarce. There seem to be no empirical studies that employ Mayer et al.’s (1995) model of trust in investigating leadership effectiveness. Dirks and Ferrin (2002) conducted a meta-analysis on trust in leadership. Much of that research investigates the dyadic relationship between a supervisor and a subordinate rather than the role of trust in specific leadership models. Dirks and Ferrin (2002) found that the research on trust and leadership tends to look at direct leaders (e.g., supervisors) as the referents of trust. They also note the many different perspectives on the construct of trust and its operationalization in leadership research. Some specific findings of that research are that trust is a determinant of the amount of cooperation to be expected between subordinate and superior (Podsakoff et al., 1990), that value congruence between subordinate and superior affected followers’ satisfaction with leadership (Sitkin and Roth, 1993; Jung and Avolio, 2000), and that antecedents to trust have included credibility (Butler, 1991) and authenticity (Avolio et al., 2004; Bass and Bass, 2008). Norman et al. (2010) found a strong impact of leader positivity (hope, efficacy, optimism, resiliency) and transparency on trust by followers.

The role of trust has been the focus of studies on TFL. Transformational leaders appear to generate more trust than transactional leaders (Den Hartog et al., 1997). Pillai et al. (1999) found a strong relationship between TFL, perceptions of fairness, and trust. The results by Gillespie and Mann (2004) confirmed the importance of trust to TFL. Zhu et al. (2013) concluded that affective trust fully mediated the relationships between TFL and the work outcomes of followers. They suggest that leaders create trusting relationships by behaving with integrity, being fair, and demonstrating their trust in followers by empowering them. Finally, Kouzes and Posner (2003) concluded that to be trusted, leaders have to be available, share personal experiences, and make connections with the experiences and aspirations of their followers.

## Mindfulness, Trust, and Leadership: an Integrative Conceptual Framework

The existing research summarized above supports the following relationships:

(1) Mindfulness → leadership
(2) Trust → leadership

We propose that, in addition to the direct impact of mindfulness on leadership, mindfulness affects leadership indirectly through an impact on trust, suggesting the following relationships:

(3) Mindfulness → trust → leadership

Specifically, we propose that leaders who are mindful exhibit attitudes and engage in behaviors that result in trust-based relationships, allowing the leader to influence others’ behaviors and, hence, to be effective.

**FIGURE 1:**
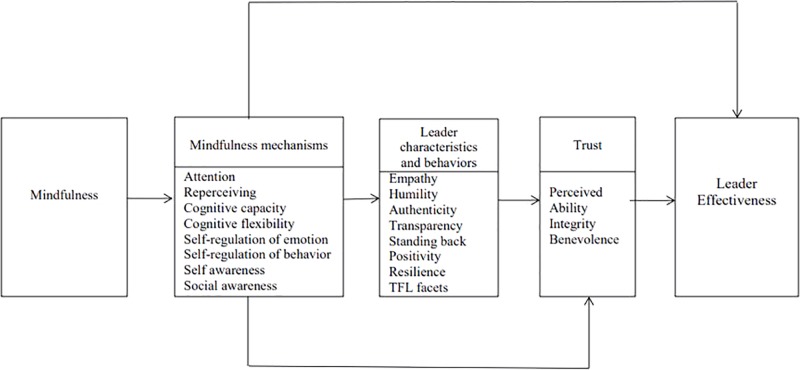
Proposed conceptual framework relating mindfulness to leader effectiveness.

The conceptual framework presented below (Figure 1) positions trust as the link between mindfulness-based leader characteristics and behaviors and leader effectiveness. Note that in addition to indirect relationships between mindfulness, trust, and leader effectiveness, we expect that mindfulness may also have a direct impact on trust and on leader effectiveness.

In the following sections, we offer theoretical reflections for the proposed linkages between mindfulness and trust in the context of leadership.

## Mindfulness and Trustworthiness of a Leader

How could mindfulness impact trust? We suggest that mindfulness contributes to a person’s trustworthiness, directly and indirectly. First, trustworthiness depends on the trustor’s perception of the trustee as competent, as having integrity, and as benevolent. How may mindfulness impact these perceptions? Second, we argue that mindfulness facilitates the empirically supported characteristics associated with effective leadership such as empathy, authenticity, and transparency and that these characteristics impact trustworthiness and trust. In the following, we reflect on these possible direct and indirect relationships for each of the components of trustworthiness.

### Mindfulness and Perceived Ability

Mindfulness is paying attention, on purpose, non-judgmentally. This is facilitated by a re-perceiving process that results in seeing reality clearly, without emotional attachment, and increases cognitive capacity and flexibility (Shapiro et al., 2006). This clarity and enhanced cognition allow leaders access to their own knowledge and skills, improving the leader’s problem-solving and decision-making. We argue that mindfulness will enhance leader ability and the followers’ perceptions of the leader as competent. This will reduce followers’ perception of uncertainty and increase their willingness to be vulnerable.

In addition, as discussed above, a leader who is authentic, transparent, positive, resilient, and provides intellectual stimulation is likely to be effective. We suggest that this is so because such a leader is more likely to be seen as competent, and, hence, more trustworthy. Authenticity and transparency allow followers to have a clear picture of the leader’s abilities – they know what he/she can do and what he/she cannot do and this reduces uncertainties and increases willingness to be vulnerable. Norman et al. (2010) explain that authentic transparent leadership represents the extent to which an individual exhibits a pattern of openness and clarity in his/her behavior toward others by sharing the information needed to make decisions, accepting others’ inputs, and disclosing his/her personal values, motives, and sentiments in a manner that enables followers to more accurately assess the leader’s competence.

Furthermore, a leader who displays higher levels of positivity (hope, efficacy, optimism, resiliency) would be seen by others as being more competent and in turn trustworthy because these components have been demonstrated to be connected to higher levels of performance (Luthans et al., 2007). In addition, a leader who is resilient will have access to his/her capabilities even under difficult circumstances and is able to perform under adverse conditions. Followers can rely on the leader’s ability, making them more willing to be vulnerable. Also, intellectual stimulation refers to leader behaviors that cultivate curiosity and creativity in followers, including behaviors that allow followers to question assumptions, reframe problems, and develop new solutions (Bass and Riggio, 2006). A leader who engages in such behaviors exposes his/her own knowledge and skills and willingness to learn – both reducing uncertainty about what the leader can do.

### Mindfulness and Perceived Integrity

Integrity is about consistency in a leader’s behavior – consistency of behavior across situations and consistency between what the leader says and does – and about the principles the leader holds. Do the principles consistently guide the leader’s behavior and are the principles aligned with the follower’s values? We suggest that self-awareness and self-regulation of emotion and behavior directly affect the extent to which a leader behaves with integrity (Shapiro et al., 2012). Parker et al. (2015) refer to self-awareness as intrapersonal awareness and internal attunement between the observing and the experiencing self. A self-aware leader knows him/herself and chooses actions based on their alignment with his/her values (self-regulation of behavior) rather than acting with automaticity based on previously established patterns of behavior or based on the emotional content of the present moment experience (self-regulation of emotion). Chances are that such self-awareness facilitates consistency in behavior and impacts trust through its effect on the followers’ perceptions of integrity.

Integrity involves the perception that the leader adheres to a set of principles that followers find acceptable. Followers, therefore, need to be aware and know what the leader’s principles are and have to have some idea about the extent to which the leader makes decisions based on this principles. Most importantly, the leader’s principles must be aligned with the follower’s principles (value congruence). Clearly, the leader’s transparency and authenticity play an essential role here. Being transparent allows followers to know the values the leader holds. An authentic leader’s values and principles are deeply rooted and not context-specific – followers can expect that the leader will always follow such principles. Verdorfer (2016) refers to this type of behavior as authentic functioning.

In addition to transparency and authenticity, the TFL component of idealized influence might be relevant. Idealized influence is considered the emotional component of leadership (Antonakis, 2012). It is associated with perceptions of integrity as followers see the leader as a role model, as someone who has very high standards of moral and ethical conduct, and as someone followers want to emulate.

### Mindfulness and Perceived Benevolence

Discussing the interpersonal benefits of mindfulness, Parker et al. (2015, p. 225) state “people high in dispositional mindfulness and experienced mindfulness meditators are often described as *warm* people, humans who are intimately in touch with the joys and sufferings of their fellow humans.” They suggest that synchronous awareness of self and others is possible because of interpersonal attunement, associated with mindfulness. Interpersonal attunement allows a focus on the internal state of another person with kindness and compassion. Elaborating on this point, Glomb et al. (2011; Siegel, 2007) note that behavioral flexibility (self-regulation of behavior) related to internal attunement – awareness of one’s own physical and emotional signals – enhances sensitivity to others without reactivity.

We expect that all of the mindfulness mechanisms directly impact this component of trust, most importantly, however, re-perceiving, social awareness, and self-regulation of emotion and behavior. Re-perceiving allows for non-identification (dis-identification), stepping back. Not feeling “threatened” opens the leader up to empathy and compassion (self-regulation of emotion). A socially aware leader knows and understands the needs, concerns, and values of his/her followers and considers them in the decisions he/she makes (self-regulation of behavior). Being in a relationship with a leader who cares about the well-being of followers reduces perceptions of uncertainty and increases the followers’ willingness to be vulnerable and their trust in the leader.

A benevolent leader is seen as a caring, warm person who is aware of and concerned with the needs and well-being of others. Empathy, humility, standing back, idealized influence, inspirational motivation, and individual consideration are expected to directly impact perceptions of benevolence.

A leader who is empathetic is willing and able to consider followers’ needs and perspectives. Verdorfer (2016, p. 952) writes “standing back from one’s own personal points of reference is a key factor in servant leadership and allows leaders to develop a sense of humbleness and acceptance and thus focus on the growth and development of others.” Thus, standing back and humility increase perceptions of benevolence and increase trust.

Idealized influence has a behavioral and an attributional element, reflecting the interactional nature of this component.Both elements capture the leader’s consideration of followers and are likely to affect perceptions of benevolence. The behavioral element represents observable leader behavior such as “the leader emphasizes the importance of having a collective sense of mission” whereas the attributional element represents the subjective, non-tangible interpretation of a leader’s behavior, e.g., the leader reassures others that obstacles will be overcome (Bass and Riggio, 2006). Also, inspirational motivation is based on the leader’s enthusiasm and optimism that creates team spirit, meaning, and challenge to the followers’ work. An inspirationally motivating leader involves followers in envisioning how things can be different and better in the future and followers know what is expected and want to meet such expectations. The potential effects of inspirational motivation on perceptions of benevolence are evident.

A leader is trusted and seen as benevolent if he/she cares about the individual follower, communicates to the followers that each of them matters. Individualized consideration is represented by interactions with followers that are personalized. The leader acknowledges the differences across followers and behaves accordingly when providing support and encouragement. Here, the leader acts as the followers’ coach and mentor who is interested in assisting them in their own development. Hence, individualized consideration is expected to strongly impact perceptions of benevolence.

## Discussion

The foundation for effective leadership is the social relationship between the leader and his/her followers (Good et al., 2016). Trust is at the core of this relationship. Leading implies influence and influence requires an individual’s willingness to be vulnerable with another person. Such willingness depends on perceptions of trustworthiness, specifically, perceptions of competence, integrity, and benevolence. In this paper, we suggest that mindfulness facilitates perceptions of trustworthiness through the effect of mindfulness mechanisms on cognitions and behaviors and the components of trustworthiness.

This paper is a theoretical reflection with the purpose of offering an initial conceptual framework for the relationship between mindfulness and leader effectiveness that highlights the importance of trust in the leader–follower relationship. Leadership is complex and critical to organizational success. Leaders are able to influence others’ behaviors and by that produce certain organizational outcomes. We suggest that mindfulness has a positive impact on trust and leader effectiveness.

Mindfulness increases a person’s ability to recognize and regulate their emotions, reduces personal identification with stimuli, allowing the person to create mental space and to simply observe what is happening without judgment and to choose a skillful response. These mindfulness effects directly impact the behaviors related to leadership. We suggested that the mindfulness-based leader characteristics and behaviors are directly related to trust and discussed how each leader characteristic and behavior may affect the components of trustworthiness. Similarly, we explained that mindfulness may directly impact trust by providing a rationale for the connection between mindfulness mechanisms and each of the components of trustworthiness.

Mindful leaders behave more consistently as they do not react automatically but respond based on their values and principles. Non-identification and emotion and behavior control result in the leader’s ability to focus on the task at hand without distractions based on fear and lack of confidence. Such “steadfastness” and enthusiasm will inspire and motivate followers.

Mindful leaders will be effective because they are able to influence others’ behavior because they are trusted. They are trusted because they engage in behaviors that increase their trustworthiness. Followers feel safe and are willing to be vulnerable in their relationship with their leader.

## Summary and Recommendations for Future Research

The primary purpose of this paper was to present an integrative conceptual framework for the relationship between mindfulness and leader effectiveness. The framework proposes direct as well as indirect relationships. Specifically, we suggest that mindfulness impacts leader effectiveness through leader characteristics and behaviors and trust. Extensive arguments and the associated relevant literature in support of the proposed linkages were presented and discussed in detail. Theoretical support for the proposed linkages was provided.

Future research might focus on exploring the proposed direct and indirect linkages between mindfulness mechanisms and leader characteristics and behaviors, between leader characteristics and behaviors and trust and the components of trust, and between mindfulness mechanisms and trust and components of trust.

This research contributes to the increasing interest in mindfulness in the workplace by offering ideas on how mindfulness improves leader effectiveness and by that the success of today’s organizations. Bringing mindfulness and leadership together holds much promise for the success of future leaders.

## Author Contributions

YS conceptualized this research and drafted the manuscript. TS contributed to the conceptualization of the paper, reviewed and edited the manuscript, and was in charge of ensuring the accuracy and completeness of the references and of meeting the formatting requirements.

## Conflict of Interest Statement

The authors declare that the research was conducted in the absence of any commercial or financial relationships that could be construed as a potential conflict of interest.
